# Comparison of drug-induced sleep endoscopy and Müller's maneuver in diagnosing obstructive sleep apnea using the VOTE classification system^☆^

**DOI:** 10.1016/j.bjorl.2016.05.009

**Published:** 2016-06-20

**Authors:** Yakup Yegïn, Mustafa Çelik, Kamïl Hakan Kaya, Arzu Karaman Koç, Fatma Tülin Kayhan

**Affiliations:** Bakırköy Dr. Sadi Konuk Training and Research Hospital, Department of Otorhinolaryngology – Head and Neck Surgery, Istanbul, Turkey

**Keywords:** Obstructive sleep apnea, Müller's maneuver, Drug-induced sleep endoscopy, VOTE classification, Apneia obstrutiva do sono, Manobra de Müller, Endoscopia com sono induzido por fármacos, Classificação VOTE

## Abstract

**Introduction:**

Knowledge of the site of obstruction and the pattern of airway collapse is essential for determining correct surgical and medical management of patients with Obstructive Sleep Apnea Syndrome (OSAS). To this end, several diagnostic tests and procedures have been developed.

**Objective:**

To determine whether drug-induced sleep endoscopy (DISE) or Müller's maneuver (MM) would be more successful at identifying the site of obstruction and the pattern of upper airway collapse in patients with OSAS.

**Methods:**

The study included 63 patients (52 male and 11 female) who were diagnosed with OSAS at our clinic. Ages ranged from 30 to 66 years old and the average age was 48.5 years. All patients underwent DISE and MM and the results of these examinations were characterized according to the region/degree of obstruction as well as the VOTE classification. The results of each test were analyzed per upper airway level and compared using statistical analysis (Cohen's kappa statistic test).

**Results:**

There was statistically significant concordance between the results from DISE and MM for procedures involving the anteroposterior (73%), lateral (92.1%), and concentric (74.6%) configuration of the velum. Results from the lateral part of the oropharynx were also in concordance between the tests (58.7%). Results from the lateral configuration of the epiglottis were in concordance between the tests (87.3%). There was no statistically significant concordance between the two examinations for procedures involving the anteroposterior of the tongue (23.8%) and epiglottis (42.9%).

**Conclusion:**

We suggest that DISE has several advantages including safety, ease of use, and reliability, which outweigh MM in terms of the ability to diagnose sites of obstruction and the pattern of upper airway collapse. Also, MM can provide some knowledge of the pattern of pharyngeal collapse. Furthermore, we also recommend using the VOTE classification in combination with DISE.

## Resumo

**Introdução:**

O conhecimento do local da obstrução e do padrão de colapso das vias respiratórias é essencial para determinar o tratamento cirúrgico e clínico corretos de pacientes com Síndrome de Apneia Obstrutiva do Sono (SAOS). Para este fim, vários testes e procedimentos de diagnóstico foram desenvolvidos.

**Objetivo:**

Determinar se a Endoscopia de Sono Induzido por Fármacos (DISE) ou Manobra de Müller (MM) seria mais bem-sucedida na identificação do local de obstrução e do padrão de colapso das vias respiratórias superiores em pacientes com SAOS.

**Método:**

O estudo incluiu 63 pacientes (52 do sexo masculino e 11 do sexo feminino) que foram diagnosticados com SAOS em nossa clínica. As idades variaram de 30 a 66 anos e a idade média foi de 48,5 anos. Todos os pacientes foram submetidos a DISE e MM e os resultados destes exames foram caracterizados de acordo com a região/grau de obstrução, bem como a classificação VOTE. Os resultados de cada teste foram analisados por nível das vias respiratórias superiores e comparados usando análise estatística (teste estatístico kappa de Cohen).

**Resultados:**

Houve concordância estatisticamente significativa entre os resultados da DISE e MM para os procedimentos que envolvem configuração anteroposterior (73%), lateral (92,1%) e concêntrica (74,6%) do véu palatino. Os resultados da parte lateral da orofaringe também estavam em concordância entre os testes (58,7%). Os resultados da configuração lateral da epiglote estavam em concordância entre os testes (87,3%). Não houve concordância estatisticamente significativa entre os dois exames para os procedimentos que envolvem a parte anteroposterior da língua (23,8%) e epiglote (42,9%).

**Conclusão:**

Sugere-se que a DISE apresenta várias vantagens, como segurança, facilidade de uso e confiabilidade, que superam a MM em termos da capacidade de diagnosticar locais de obstrução e o padrão de colapso da via respiratória superior. O MM pode também fornecer algum conhecimento sobre o padrão de colapso da faringe. Além disso, recomendamos o uso da classificação VOTE em combinação com DISE.

## Introduction

In 1973, Guilleminault first described obstructive sleep apnea (OSA) as a syndrome characterized by recurrent episodes of sleep apnea and hypopnea caused by repetitive upper airway (UA) collapse. OSA often results in decreased oxygen levels in blood and arousal from sleep.^1^ Obstructive Sleep Apnea Syndrome (OSAS) may induce excessive daytime somnolence, morning headaches, poor concentration, cardiopulmonary and cardiovascular diseases, and a lower quality of life.^2^, ^3^

Polysomnography, first described in 1965 by Gastaut, is utilized to diagnose and assess the severity of OSAS.^4^ However, knowledge of the site of obstruction and the pattern of airway collapse is essential for determining correct surgical and medical management of patients with OSAS. To this end, several diagnostic tests and procedures have been developed. Fiber-optic nasal endoscopy was first utilized by Weitzman and Hill to diagnose patients with OSAS.^5^, ^6^ In 1978, Müller's maneuver (MM) was introduced by Borowiecki et al.^7^ to determine sites of airway collapse in patients with OSAS. Sher et al.^8^ suggested that MM is beneficial for identifying the correct surgical procedure in patients with OSAS. Previous studies have determined that the physiology of the upper airway is different during wakefulness and sleep. In 1991, Croft and Pringle introduced sleep endoscopy, which was an endoscopic examination performed during drug-induced sleep to visualize upper airway collapse.^9^

In this study, we compared two methods, DISE and MM, regarding their ability to identify the site and degree of upper airway collapse, and characterized according the VOTE classification.

## Methods

We conducted a retrospective review of data collected from November 2013 to August 2014 at the our hospital within the Department of Otolaryngology Head and Neck Surgery. There were 63 patients included in the study, 52 males and 11 females, with an average age of 48.5 ± 8.9 years old (range, 30–66). We included patients with an apnea–hypopnea index greater than 5, as determined by on overnight sleep study. Patients were excluded if they had any of the following characteristics: an apnea–hypopnea index lower than 5, less than 18 years old, body mass index (BMI) greater than 40, history of previous sleep surgery, American Society of Anesthesiologists (ASA) grade 3–4, and patients who refused surgical therapy. Each patient was evaluated based on the Epworth Sleepiness Scale (ESS), apnea–hypopnea index (AHI), BMI, and neck circumference.

For all OSAS patients in this study, MM and DISE were performed by the same surgeon. Topical nasal decongestant and topical anesthetic (10% lidocaine) were applied to both nasal cavities. Patients were placed in a supine position on the operating room table with the lights dimmed. A flexible fiber-optic laryngoscope was passed through the anesthetized nasal cavity into the larynx and observations were digitally recorded. The pattern, site, and degree of upper airway collapse were characterized according to the VOTE classification. The following upper airway sites were evaluated: velum, oropharynx lateral wall, tongue, and the epiglottis. All patients were provided the necessary information to perform the MM, which the patients then performed by maintaining maximal inspiration with an open glottis against closed oral and nasal airways. The same maneuver was performed for each upper airway level. The degree of upper airway collapse was divided into three categories: total, partial obstruction, and no obstruction, all according to the VOTE classification. DISE was also performed on all patients in a silent operating room with each patient in a supine position. First, atropine (0.5 mg/kg) was applied to reduce upper airway secretion, and then topical nasal decongestant and topical anesthetic (10% lidocaine) were applied to both nasal cavities. Throughout this procedure, oximetry and cardiac rhythms were monitored by an anesthetic team and supplemental oxygen was administered by a blow-by facemask (or when necessary, a nasal cannula). Sedation was achieved using an infusion of a standard propofol titration protocol beginning at a rate of 50–75 mcg/kg/min. For patients who had snoring or obstructive apneas, we passed a flexible fiber-optic laryngoscope through the anesthetized nasal cavity. Following DISE, we utilized the VOTE classification system to evaluate the upper airway collapse. For all patients, both endoscopic procedures were performed by the same surgeon. All patients were well informed and provided written informed consent. The protocol for this study was approved by the same hospital's local ethics committee (Ethical Committee number 2014/164).

### Statistical analysis

The Number Cruncher Statistical System (NCSS) 2007 Statistical Software (UT, USA) was used for statistical analyses. Data were evaluated using descriptive statistical methods (e.g., mean, standard deviation, median, interquartile range). The results of both procedures were statistically analyzed using Cohen's kappa statistical test. Results with a *p*-value < 0.05 were considered statistically significant.

## Results

The mean AHI for all of the patients was 33.8 ± 20.5 events/h and ranged from 5 to 94.6 events/h. The mean BMI was 29.2 ± 4.3 kg/m^2^ and ranged from 19.6 to 38.3 kg/m^2^. The mean neck circumference was 41 ± 3.1 cm with values ranging from 33 to 46 cm. The mean ESS was 9.5 ± 6.4 with values ranging from 0 to 24. The results of all of these tests are listed in Table 1.

**Table 1:** Patient demographics.

Characteristic	Average
Age, yr ± SD	48.5 ± 8.9
Male, *n* (%)	52 (82.5%)
AHI, events/h ± SD	33.8 ± 20.5
NC, cm ± SD	41.0 ± 3.1
ESS, *n* ± SD	9.5 ± 6.4
Mallampati 3–4, *n* (%)	59 (93.7%)

SD, standard deviation; AHI, apnea–hypopnea index; NC, neck circumference; ESS, Epworth Sleepiness Scale.

**Figure 1:**
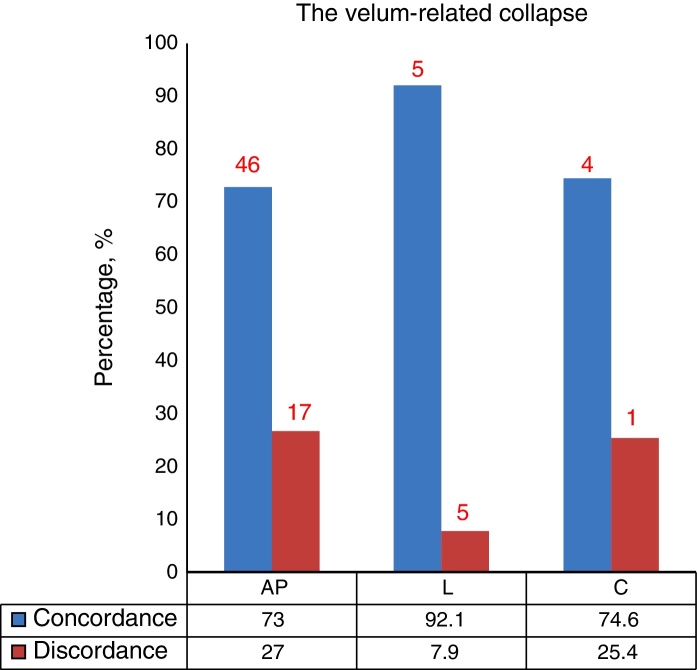
Concordance of the diagnosis of velum-related collapse in anteroposterior, lateral, and concentric configurations (AP, anteroposterior; L, lateral; C, concentric).

Among the 63 patients, 30 patients lying in an anteroposterior configuration, 5 patients in a lateral configuration, and 27 patients in a concentric configuration had velum-related obstruction as observed by both MM and DISE. For each of these configurations, there was significant concordance in the diagnosis of velum-related obstruction between the two methods (anteroposterior, 73%; *κ* = 0.55, *p* < 0.05), lateral, 92.1%; *κ* = 0.348, *p* < 0.05), and concentric, 74.6%; *κ* = 0.555, *p* < 0.05) (Fig. 1).

**Figure 2:**
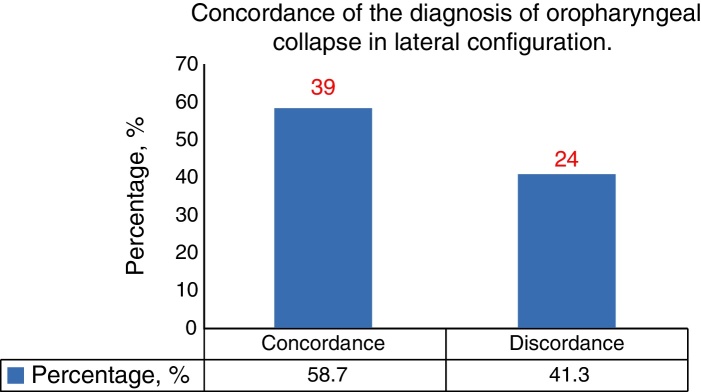
Concordance of the diagnosis of oropharyngeal collapse in lateral configuration.

Both endoscopic techniques identified 50 patients as having oropharynx-related obstruction in the lateral configuration (58.7%; *κ* = 0.414, *p* < 0.05); none had it in the anteroposterior or concentric configurations according to either procedure (Fig. 2).

**Figure 3:**
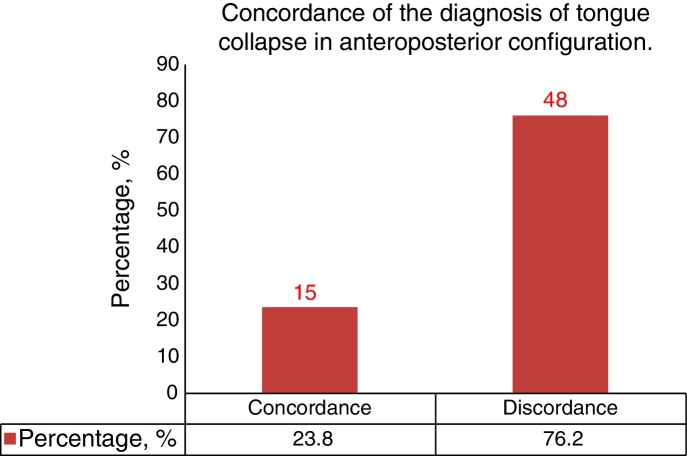
Concordance of the diagnosis of tongue collapse in anteroposterior configuration.

At the tongue level, 20 patients in the anteroposterior configuration were diagnosed with severe upper airway collapse when examined via MM compared to 51 patients diagnosed via DISE. This reveals a lack of concordance in the diagnosis of severe tongue-related collapse between the two methods (76.2%; *κ* = 0.026, *p* > 0.05) (Fig. 3).

At the level of the epiglottis (anteroposterior configuration), we observed severe collapse in 11 patients examined via MM compared to 39 patients when examined by DISE, demonstrating a lack of concordance between the two methods (57.1%; *κ* = 0.107, *p* > 0.05).

**Figure 4:**
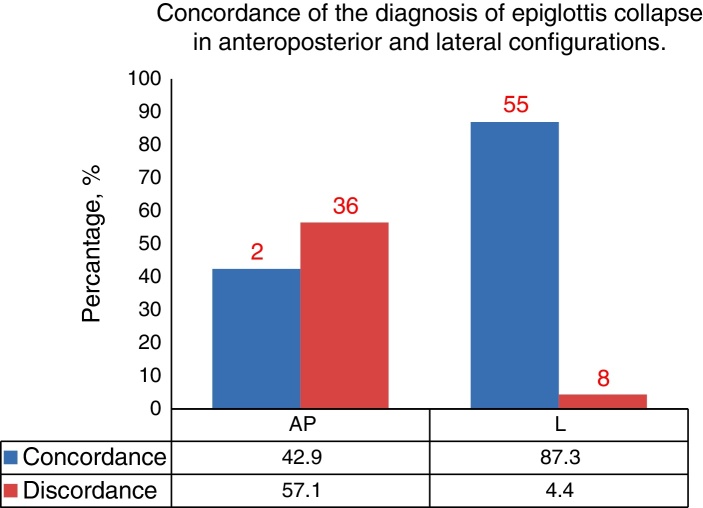
Concordance of the diagnosis of epiglottis collapse in anteroposterior and lateral configurations (AP, anteroposterior; L, lateral).

In contrast, in the lateral configuration, 5 patients examined via MM compared to 9 patients examined via DISE were diagnosed with severe upper airway collapse, demonstrating significant concordance (87.3%; *κ* = 0.383, *p* < 0.05) (Fig. 4).

## Discussion

Identification of the site and pattern of upper airway collapse is essential to accurately prescribe therapeutic approaches for patients with OSAS. Furthermore, patients are likely to have different sites and patterns of upper airway collapse, as well as a range of severities of the disease. Therefore, identifying an accurate surgical treatment for OSAS patients will reduce unnecessary expenses.^10^, ^11^

Several methods have been utilized to diagnose the presence of severe level-specific upper airway collapse.^12^, ^13^ In 1977, Weitzman et al.^5^ introduced endoscopic examination for identifying the site of upper airway collapse in OSAS patients. One technique that has readily been utilized in clinical settings is MM. MM can be performed in patients with OSAS to determine the site of obstruction in the upper airways since it is a cheap and easily performed method that can provide valuable information. In the present study, MM was performed in a supine position different from previous studies. The mechanism responsible for the worsening of OSA in the supine position remains unclear. Most likely it relates to the effect of gravity on the size or shape of the upper airway. A smaller pharyngeal airway in the supine position, making it more vulnerable to collapse, is an intuitive explanation. However, studies on the pharyngeal size between the two postures are inconsistent.^2^, ^3^ Until now, the effect of posture on the upper airway during sleep in OSA patients remains largely unclear. To our knowledge, there is no reported study that focused on the interaction between the supine and seated positions in MM. Therefore, MM was animadverted for being subjective and for yielding different results when applied by different examiners.^10^, ^12^ Terris et al.^14^ reported that despite differences in the experience of the examiners, it had a high inter-examiner concordance, and was a valuable examination. However, one limitation is that it is performed in an awake state, and the severity of collapse differs between a conscious and unconscious state, likely due to differences in the tone of the upper pharyngeal muscle. For this reason, several studies have instead focused on endoscopy during sleep. Borowiecki et al.^7^ did not observe any obstruction at the level of the larynx when 10 patients with sleep apnea and hypersomnia were asleep. Croft and Pringle^15^ introduced DISE in 1991. This type of endoscopy directly visualizes upper airway collapse in patients lying in a supine position.

DISE has frequently been utilized in clinical settings. However, our understanding of the advantages associated with DISE have been clouded due to the multitude and complexity of classification systems used to compare results among studies.^15^, ^16^, ^17^, ^18^

Several studies have compared the results obtained from using MM to those reported using DISE. Soares et al.^19^ reported that these endoscopic methods produced similar diagnoses of retropalatal collapse in 53 OSAS patients. However, DISE indicated in a much higher incidence of severe retrolingual collapse compared to MM. Furthermore, Cavaliere et al.^20^ demonstrated that using MM is more likely to hypothesize the diagnosis of laryngeal obstruction in OSAS patients. Overall, data on the comparison of DISE with MM in terms of the identification of the site and pattern of upper airway collapse remains sparse.

In this study, we compared the diagnosis of the sites and degree of upper airway collapse, according to the VOTE classification system, between these two endoscopic methods. Although MM is a dynamic test, whereas VOTE is a static classification for identification of the site and pattern of upper airway collapse, comparison of results this different methods seems not be ideal. Therefore, no any dynamic classification system was used for identification of the site and pattern of upper airway collapse in literature. The VOTE classification system provides valuable knowledge of upper airway obstruction statically, and only the obstruction can be determined as anteroposterior, lateral or concentric configurations. Therefore, it may be inadequate for exact identification of upper airway collapse. The VOTE classification system contains the most commonly involved structures, containing the degree and configuration of the obstruction related to them. Although the VOTE classification system not reflects the degree of upper airway obstructive events exactly, it provides valuable knowledge's for identification of the site and pattern of upper airway collapse. No consensus has yet emerged regarding the gold standard classification system for identification of upper airway collapse. Furthermore, the use of a universal scoring system can facilitate the scientific assessment of studies conducted in single centers, as well as multicentric studies, allowing comparison of results.^17^, ^20^ Lack of data hinders the resolution of several controversial issues. VOTE classification is a qualitative assessment method measured as the degree of upper airway obstructive events, which focuses on primary structures that contribute to upper airway collapse and their relationship to the severity of the collapse.^20^, ^21^ The severity associated of collapse and its classification is contingent on a surgeon's experiences and reliability.^17^ Upper airway obstruction is classified as none, partial, and complete, which is general but useful to guide the treatment options for OSAS patients because it is difficult to determine an exact percentage of obstruction in patients.

The uvula and soft palate are the main factors involved in the collapse of the velopharyngeal level in patients with OSAS.^16^, ^22^ The obstruction at this level can occur via collapse in an anteroposterior, lateral, or concentric configuration.^21^ In our study, there was statistically significant concordance between the two endoscopic procedures for all configurations with regards to velopharyngeal-related obstruction.

Tonsils, lateral pharyngeal wall tissues that consist of musculature, and adjacent parapharyngeal fat pads all contribute to the collapse of the oropharyngeal level in patients with OSAS.^12^, ^13^ The obstruction at this level can occur via collapse in the lateral and concentric configuration.^23^ We observed statistically significant concordance in the diagnosis of oropharyngeal-related obstructions in the lateral configuration between the two methods.

Obstruction related to the tongue is a common finding in patients with OSAS and is mainly identified in an anteroposterior configuration.^23^ Dilation in the muscle tone of the tongue is more pronounced in patients with OSAS.^22^, ^23^, ^24^ In our study, the presence and degree of tongue obstruction differed by method, with MM indicating severe collapse in 20 patients and DISE indicating severe collapse in 51 patients in the anteroposterior configuration at the tongue level. In the present study, the presence of tongue obstruction was higher than previous studies in literature. Confounding variables that were addressed include the deep sedation, higher Mallampati scores, oversensitive observation and prolonged examination. In the present study, we did not use bispectral index monitoring to determine the level of sedation objectively. Also, we did not assert that the similar level of sedation was provided in all patients. Secondly, there is heterogeneity between the present study and published studies with regard to Mallampati scores. Thirdly, experience of surgeon can affect the identification of the site and pattern of upper airway collapse. Overall, several variables are different between OSA patients including such as age, body mass index, prior surgeries, cephalometric variables, gender, race which contribute to heterogeneity between the present study and published studies.

We did not observe significant concordance in the incidence of severe epiglottis-related collapse in the anteroposterior configuration using MM compared to DISE, but did so in the lateral configuration. Further studies with larger sample sizes are necessary to support the high correlations observed in this study between MM and DISE in diagnosing epiglottis-related obstruction in the lateral configuration.

In a prospective study of Gregorio et al.^1^ reported that more retroglossal obstructions were detected during sleep endoscopy compared to MM. On the other hand, DISE is not a natural sleeping state. In natural sleep, there is a reduction in genioglossus muscle tone during NREM and REM sleep that is more pronounced in OSA than normals. During propofol unconscious sedation, reductions in genioglossus tone also occur and can contribute to tongue base collapse.

Limitations of this study the sample size, a lack of the level of sedation and a lack of randomization. If the study design was randomized study with larger sample sizes, the study may be more valuable.

## Conclusion

In conclusion, we observed statistically significant concordance in the diagnoses of obstructions related to the velum and oropharynx between MM and DISE. In contrast, we did not observe this concordance between techniques when identifying obstruction related to the tongue and epiglottis. However, MM can be performed in patients with OSAS to determine the site of obstruction in the upper airways since it is a cheap and easily performed method that can provide some knowledge of the pattern of pharyngeal collapse. We suggest that DISE is a valid, safe, easy-to-use method for identifying the severity of upper airway collapse at different airway levels. We also recommend using the VOTE classification in combination with DISE.

## Conflicts of interest

The authors declare no conflicts of interest.
